# Endoscopic Repair of Tracheoesophageal Fistula with Vascular Embolization Plug

**DOI:** 10.3390/diagnostics15192529

**Published:** 2025-10-07

**Authors:** Predrag Sabljak, Ognjan Skrobic, Milica Mitrovic-Jovanovic, Ivan Vukasinovic, Aleksandra Djuric-Stefanovic, Anja Zugic, Dario Potkonjak, Marija Đorđevic, Keramatollah Ebrahimi

**Affiliations:** 1 Clinic for Digestive Surgery, University Clinical Centre of Serbia, Koste Todorovica Street, No. 6, 11000 Belgrade, Serbiadariopotkonjak10@gmail.com (D.P.); marijadjordjevic7@outlook.com (M.Đ.); 2 Faculty of Medicine, University of Belgrade, Dr Subotica No. 8, 11000 Belgrade, Serbia; 3 Center for Radiology and Magnetic Resonance Imaging, University Clinical Centre of Serbia, Pasterova No. 2, 11000 Belgrade, Serbia

**Keywords:** tracheoesophageal fistula, endoscopic vascular plug repair

## Abstract

Aerodigestive fistulas represent a major challenge in clinical practice. This problem is burdened with severe morbidity and mortality, despite recent advantages in endoscopic endoluminal repair techniques. Special problems are fistulas localized higher, engaging the proximal esophagus and trachea, which in adults most often result from post-intubation injury. Surgery is generally demanding and reserved for the patients in whom other, less invasive options fail. Hereby, we present a case of post-intubation tracheoesophageal fistula, successfully treated with endoscopic vascular plug placement.

**Figure 1:**
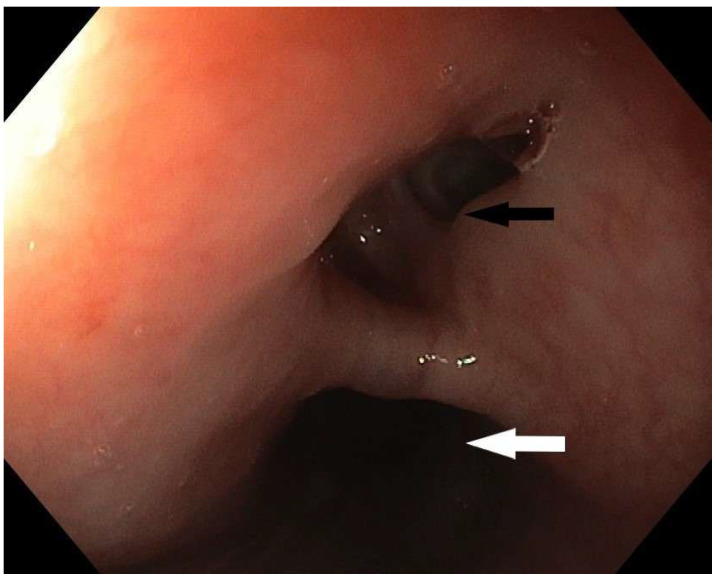
Upper gastrointestinal endoscopy in a female patient, 60 years old, revealed the presence of a fistula opening 20 cm from the incisors. This image shows the opening of the fistulous canal (black arrow) and the lumen of the esophagus (white arrow). The patient was admitted to the regional hospital for laparoscopic cholecystectomy. The prior day, ERCP procedure was performed, and a gallstone was removed from the main hepatic duct. Immediately after the initiation of the surgical procedure, and creation of the pneumoperitoneum, the patient experienced dramatic respiratory instability, a drop in oxygen saturation, and extreme gastric air dilatation, so the surgical procedure was abandoned. Liquid green output, highly suspicious for gastric content, was observed from the endotracheal tube. An emergency CT was performed, confirming the diagnosis of tracheoesophageal fistula. Two days later, the patient was admitted to the Intensive Care Department of the Clinic for Digestive Surgery, University Clinical Center of Serbia. The patient was intubated, sedated, and on continuous vasopressor stimulation. Tracheoesophageal fistula (TEF) in adults is usually an acquired condition, which can be further divided into benign and malignant TEF. Benign fistulas are mostly iatrogenic (post-intubation, post-surgery, post-radiation) [1]. Post-intubation trauma is responsible for approximately 75% of benign tracheoesophageal fistula cases [2]. The diagnostic modalities include contrast radiography, CT imaging, bronchoscopy, and upper gastrointestinal endoscopy. Upper GI endoscopy clearly shows a defect in the esophageal wall with a formed fistulous tract which was confirmed by a CT scan showing a pathological communication between the anterior wall of the esophagus and the initial part of the left bronchus. The fistulous tract has a maximum lumen width of about 6 mm (Figure 2). MDCT revealed esophago-tracheal fistula, with the ostium on the esophagus 11 mm of width, 6 mm opening on the tracheal wall, and fistulous tractus 6 mm in diameter.

**Figure 2:**
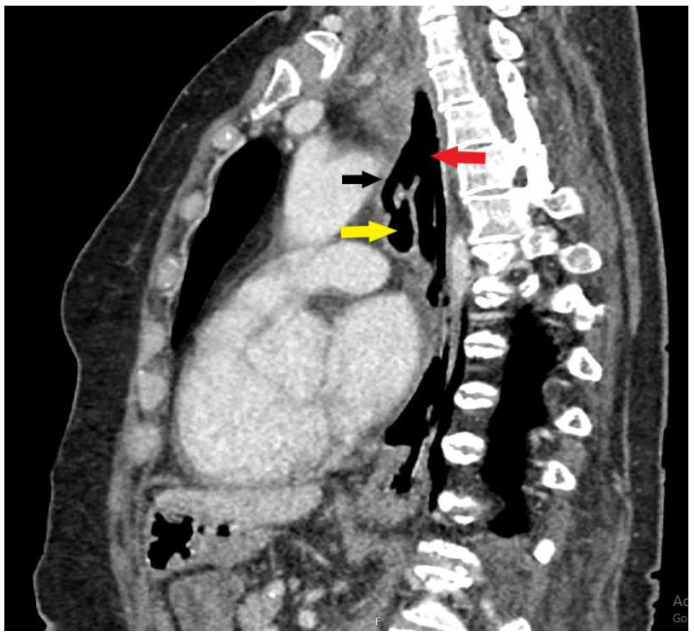
A sagittal CT scan shows interluminal communication (black arrow) between the lumen of the esophagus (red arrow) and the trachea (yellow arrow). There were also signs of aspiration pneumonia bilaterally and segmental pulmonary artery embolism (Figure 3).

**Figure 3:**
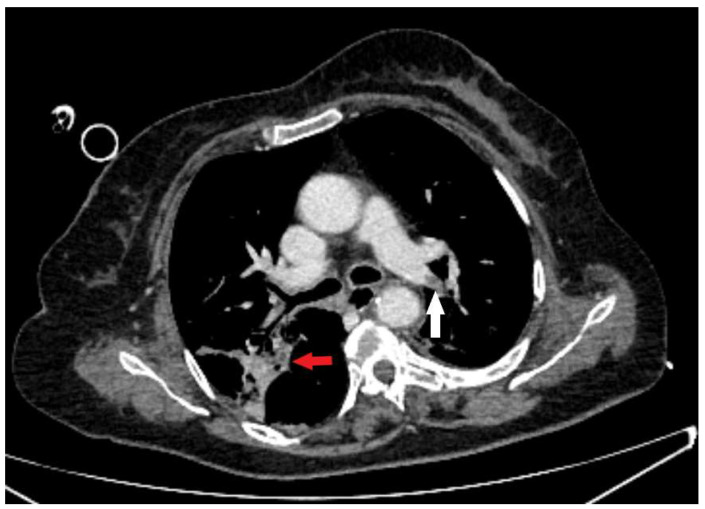
An axial CT scan demonstrates intraluminal defect in the postcontrast opacification of the lobar branch of the left pulmonary artery (white arrow), which corresponds to pulmonary thromboembolism with present consolidative parenchymal zones consistent with aspiration pneumonia (red arrow).

**Figure 4:**
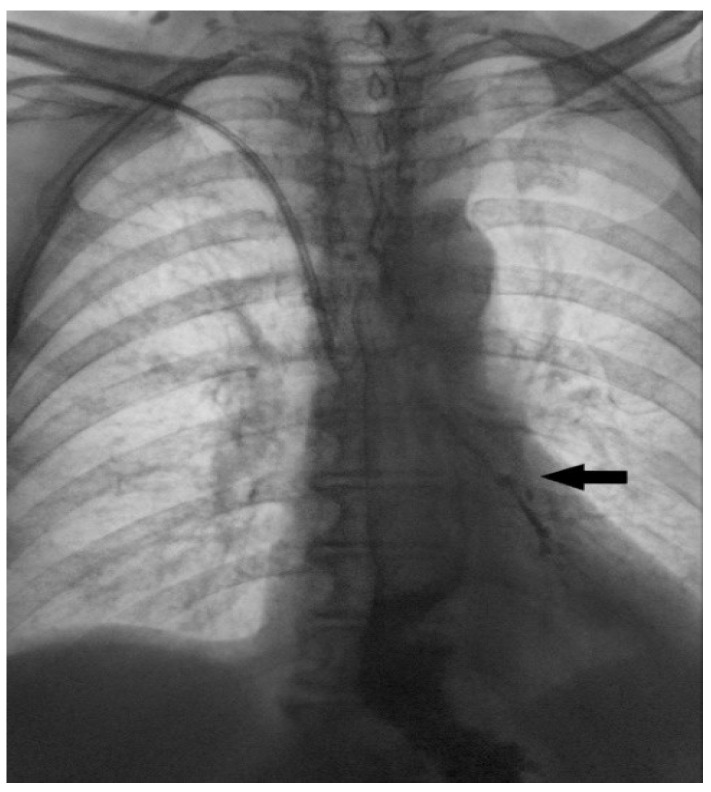
Contrast radiography is a very useful method in terms of establishing this diagnosis. The appearance of contrast in the lumen of the tracheobronchial tree (black arrow) is a direct sign of the fistulization of these structures within the esophagus. This image shows a clear esophagobronchial leak (fistula), which initiated a pronounced cough during the patient’s examination. Tracheobronchoscopy was also performed, showing an opening on the left side of tracheal bifurcation. In the further course of treatment, percutaneous endoscopic gastrostomy (PEG) was performed, and feeding via PEG was initiated. The patient responded well to antibiotics treatment, and was extubated 7 days after the admission to the intensive care unit. Surgery usually represents the primary treatment modality for acquired TEF. However, when surgery is contraindicated or cannot be performed as a primary solution, non-surgical treatment options may be explored. These can serve as a bridge to definitive surgical repair or, in cases of smaller fistulas, may even offer a definitive therapeutic option [3]. Esophageal stenting is an attractive, minimally invasive approach, used in treating predominantly malignant TEFs but also benign etiology [4]. Studies show a great decrease in respiratory complications, length of hospital stay, and overall quality of life, as well as better performance rate in these patients [5,6]. A retrospective study, comparing 22 patients with benign TEF treated with esophageal stenting showed successful closure of the fistula, obtained in 45% of cases. However, if the fistula is located in the proximal third of the esophagus, stenting may not be feasible [7]. Other authors utilize esophageal stenting together with airway stenting. This kind of approach may be more effective than esophageal stenting alone; however, the friction between the two stents can often damage the tissue between the trachea and esophagus. As a result, this combined approach is reserved only in selected cases [8]. After stabilizing the patient’s general condition and conducting a detailed case analysis, experts in this field decided to use a minimally invasive treatment option provided by interventional vascular radiology and attempt to close the fistulous tract by placing an IMPEDE Embolization plug coil to seal the opening in the wall of the esophagus and the trachea. In consultation with the interventional radiologist, the procedure was precisely planned. First, a flexible endoscope was introduced into the esophagus and a guidewire was placed from the lumen of the esophagus into the fistulous tract and a bronchoscope was used to confirm that the end of the guidewire was in the lumen of the trachea. Then, two IMPEDE Embolization plugs were successively placed one on top of the other, to fill the fistulous canal.

**Figure 5:**
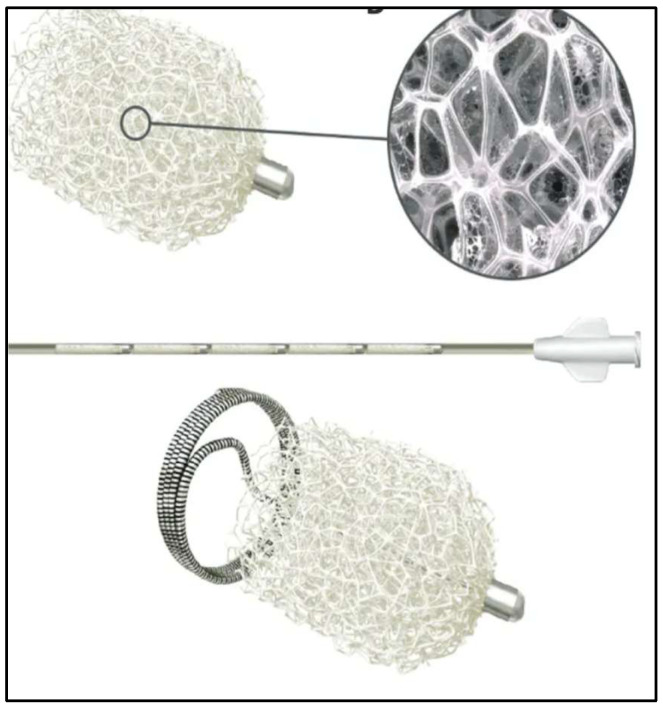
IMPEDE Embolization plug coil [9] consists of a new smart polymer and an anchoring coil that ensures the stability of the device.

**Figure 6:**
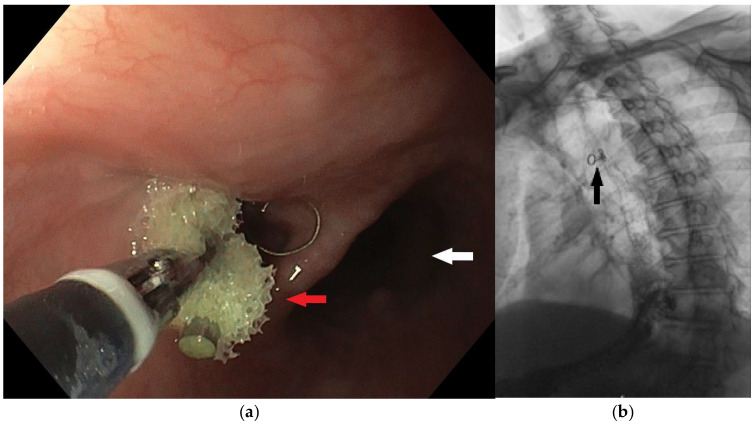
A metal coil (IMPEDE Embolization plug) was placed into the fistula tract (red arrow) by an interventional radiologist. The white arrow shows the lumen of the esophagus (**a**). The correct localization of the placed coil was confirmed by a chest x-ray (black arrow) (**b**).

**Figure 7:**
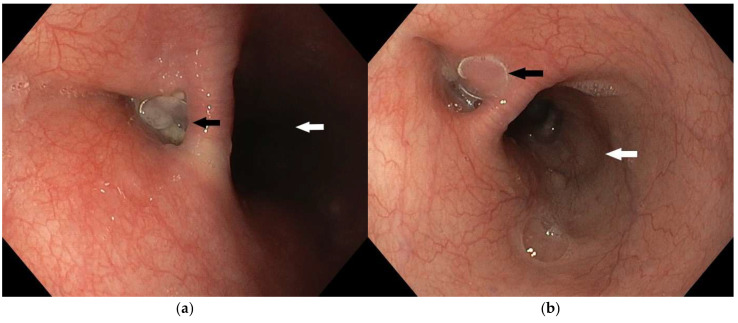
In further monitoring the patient’s condition and implementation of the placed coil (black arrows), gradual signs of epithelialization were observed. On the fifth day (**a**) and the fourteenth day (**b**) after the procedure endoscopic check/up was performed, an acceptable position of the coil (black arrow) and fistula closure were revealed. The lumen of the esophagus is indicated by a white arrow.

**Figure 8:**
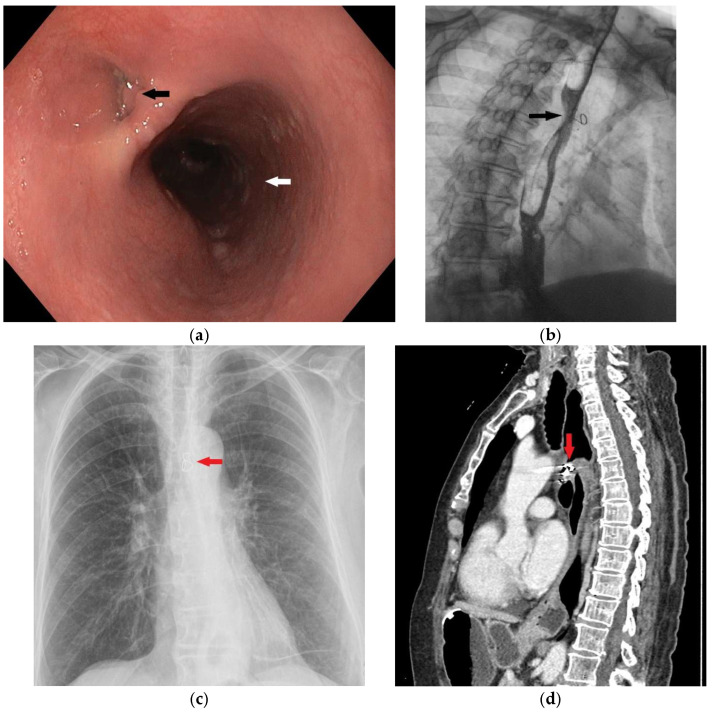
In order to thoroughly control and monitor the unusual procedure, all necessary diagnostic methods were performed three months after and the correct position and clear epithelialization of the fistulous tract were confirmed, first by upper GI endoscopy (**a**) (black arrow showing the obliterated lumen of the opening in the esophageal wall) and then by contrast X-ray examination (**b**), where no extraluminal gastrografin enema was observed (black arrow). An upright posteroanterior (PA) chest radiograph (**c**) shows the bent wire component of the coil in a correct position (red arrow), while a CT examination (**d**) confirms the correct localization of the coil within the fistulous tract with completely closed openings in the wall of the esophagus and trachea (red arrow). At that time, percutaneous gastrostomy was removed, and three months after a planned laparoscopic cholecystectomy was performed without intraoperative or postoperative complications. In the recent literature, off-label occluding devices have been used in treating TEF, usually cardiac septal defect occluders. These kinds of devices have been utilized in selected cases, especially in benign TEF and broncho-esophageal fistula [10]. However, its use has been associated with great airway complications, such as airway obstruction mainly due to mucus build up and granulation tissue. In some cases, a denovo fistula could arise due to the erosive effect that the device could have on the tissue [11]. Other approaches have used an Acell matrix (a decellularized porcine urinary bladder matrix), which has been used in treatment of benign TEFs due to its ability to enhance the healing process. Complete closure has been reported in some cases within 10 days [12]. Nevertheless, these approaches remain rarely used due to limited evidence and lack of validation in larger patient groups. Tissue adhesives such as fibrin and cyanoacrylate glue have represented another modality that can be utilized alone or in addition to other occluding devices [13]. The application of these adhesive glues is usually reserved for the pediatric population but has been found in the treatment of the adult population [14]. These agents show a better success in treating more proximal lesions, where the applicability of stents is limited. Having said that, greater outcomes using these agents are usually seen in smaller fistulae and could offer a safer approach in patients with contraindication for severe surgical procedures [15,16]. Endoluminal vacuum-assisted closure (EVAC) therapy has recently emerged as a potential option for TEF management. While promising, EVAC use in TEF remains largely limited to case reports and small series [17].

**Figure 9:**
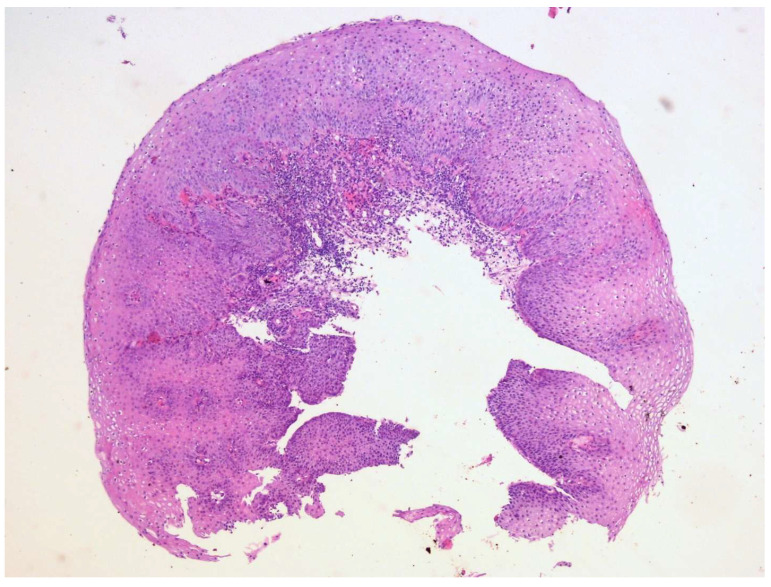
During control endoscopy, biopsies were taken from the tissue covering the coil itself. A regenerative zone of the mucosa was observed with reactive changes in the squamous cell epithelium, and another sample showed remnants of granulation tissue (HE, magnification 10×). The patient was discharged one month after the coil placement, in good general condition, with normal peroral feeding. No symptoms or complications related to the fistula were present at the moment of discharge from the hospital. In conclusion, the management of TEF requires prompt intervention to prevent serious complications that this condition carries along. Surgical repair remains the gold standard and is often the most effective treatment, particularly for larger or complex fistulas. However, in cases where surgery is contraindicated or not feasible, a variety of non-surgical options, including stenting, endoscopic techniques, tissue adhesives, and occluding devices are available. Given the limitations and variable success rates of these alternatives, treatment should be carefully individualized based on patients’ clinical condition, fistula characteristics, and overall prognosis.

## Data Availability

The original contributions presented in this study are included in the article. Further inquiries can be directed to the corresponding author.
